# False Teeth

**Published:** 1873-09

**Authors:** 


### FALSE TEETH.

The *British Medical Journal* tells the following stories of Cuvier and Lord Brougham :

Cuvier, impatient at the interruptions of that perpetual interrupter, M. Glais-Bizoin, in the National Assembly, rose so impatiently to answer him, that he jerked his teeth out on the floor, and stooping not less precipitately to pick them up, fell head fore-most, and struck his head against the floor so heavily as to give rise to the illness which proved fatal to him. M. Glais-Bizoin, then a very young man, promised himself that he would abstain from his fatal habit of incessantly interrupting ; but he was incorrigible.

Lord Brougham, in the course of the proceedings of a great meeting of the Social Science Association, of which he was president, was stopped in the middle of a speech by his teeth falling out. After groping on the floor, and on presently resuming his speech, he made the best of the incident by observing, "Our teeth are sources of trouble from infancy to old age."

